# Collective Resistance in Microbial Communities by Intracellular Antibiotic Deactivation

**DOI:** 10.1371/journal.pbio.2000631

**Published:** 2016-12-27

**Authors:** Robin A. Sorg, Leo Lin, G. Sander van Doorn, Moritz Sorg, Joshua Olson, Victor Nizet, Jan-Willem Veening

**Affiliations:** 1 Molecular Genetics Group, Groningen Biomolecular Sciences and Biotechnology Institute, Centre for Synthetic Biology, University of Groningen, Groningen, The Netherlands; 2 Department of Pediatrics University of California, San Diego, La Jolla, California, United States of America; 3 Groningen Institute for Evolutionary Life Sciences, University of Groningen, Groningen, The Netherlands; 4 Skaggs School of Pharmacy and Pharmaceutical Sciences, University of California, San Diego, La Jolla, California, United States of America; 5 Department of Fundamental Microbiology, Faculty of Biology and Medicine, University of Lausanne, Lausanne, Switzerland; University of Oxford, United Kingdom

## Abstract

The structure and composition of bacterial communities can compromise antibiotic efficacy. For example, the secretion of β-lactamase by individual bacteria provides passive resistance for all residents within a polymicrobial environment. Here, we uncover that collective resistance can also develop via intracellular antibiotic deactivation. Real-time luminescence measurements and single-cell analysis demonstrate that the opportunistic human pathogen *Streptococcus pneumoniae* grows in medium supplemented with chloramphenicol (Cm) when resistant bacteria expressing Cm acetyltransferase (CAT) are present. We show that CAT processes Cm intracellularly but not extracellularly. In a mouse pneumonia model, more susceptible pneumococci survive Cm treatment when coinfected with a CAT-expressing strain. Mathematical modeling predicts that stable coexistence is only possible when antibiotic resistance comes at a fitness cost. Strikingly, CAT-expressing pneumococci in mouse lungs were outcompeted by susceptible cells even during Cm treatment. Our results highlight the importance of the microbial context during infectious disease as a potential complicating factor to antibiotic therapy.

## Author Summary

Antibiotic-resistant bacterial infections are on the rise and pose a serious threat to society. The influence of genetic resistance mechanisms on antibiotic therapy is well described. However, other factors, such as epigenetic resistance or the impact of the environment on antibiotic therapy, are less well understood. Here, we describe and characterize a mechanism of noninherited antibiotic resistance that enables the survival and outgrowth of genetically susceptible bacteria during antibiotic therapy. We show that bacteria expressing the resistance factor chloramphenicol (Cm) acetyltransferase (CAT) can potently deactivate Cm in their immediate environment. The reduced Cm concentration then allows for the outgrowth of genetically susceptible bacteria in the same environment. Mathematical modeling demonstrates the presence of a parameter space in which stable coexistence between Cm-susceptible and -resistant bacteria is possible during antibiotic therapy, which we validated using single-cell analyses. Strikingly, mixed culture experiments in which mice were infected with both Cm-susceptible and -resistant pneumococci revealed that Cm-sensitive “freeloader” bacteria even outcompeted resistant bacteria during antibiotic therapy. Together, we show that the microbial context during infection is a potential complicating factor to antibiotic treatment outcomes.

## Introduction

Antibiotics are indispensable for fighting bacterial infections. Yet the rapid emergence of resistance during the last decades renders current drugs increasingly ineffective and poses a serious threat to human health [1]. Drug action and bacterial resistance mechanisms are well understood in population assays of isogenic cultures in vitro. However, ecological factors and cell physiological parameters in natural environments influence the impact of antibiotics [2,3]. *Streptococcus pneumoniae* (pneumococcus) is an important human pathogen that resides in complex and dynamic host environments. The bacterium primarily populates the nasopharynx of healthy individuals, together with numerous commensal microbiota, and often alongside disease-associated species, including *Staphylococcus aureus*, *Moraxella catarrhalis*, and *Haemophilus influenzae* [4–6].

While an individual pneumococcal cell competes for limited resources with all other bacteria present in the niche, it may also benefit from a community setting. In a collective effort, bacteria become recalcitrant to antibiotics when forming biofilms that represent a physical constraint for drug accessibility [7,8]. Additional population-based survival strategies involve the phenotypic diversification of an isogenic population, either to preadapt for environmental changes (bet-hedging) or to enable division of labor [9]. Because the impact of most antibiotics is growth rate dependent [10–12], a bifurcation into growing and nongrowing cells increases the drug tolerance for the latter fraction, commonly referred to as persisters [13,14]. Cell-to-cell communication represents another way to react to antibiotic inhibition by allowing bacteria to coordinate a common response; *S*. *pneumoniae*, for example, activates the developmental process of competence whereupon it may acquire resistance [15–17]. A quorum-sensing mechanism that compromises antibiotic effectiveness was also found in evolved *Escherichia coli* cultures, in which cells of increased resistance induce drug efflux pumps in susceptible cells via the signaling molecule indole [18].

As an alternative to reduced drug susceptibility, bacteria can also clear lethal doses of antibiotics from their environment. High cell densities and thus the presence of many drug target sites may be sufficient to lower the concentration of active compound by titration of free drug molecules [19]. Furthermore, antibiotic degradation via β-lactamase enables growth not only of resistant cells but also of susceptible cells in their vicinity [20–22], even across species, as demonstrated for amoxicillin-resistant *H*. *influenzae* and susceptible *S*. *pneumoniae* [23,24]. This mechanism is of direct relevance to clinical medicine and is alternatively referred to as passive or indirect resistance (from the perspective of susceptible cells) or collective resistance (from the perspective of mixed populations) [25].

Here, we describe another mechanism by which bacteria survive antibiotic therapy without obtaining genetic resistance, with the example of the bacteriostatic antibiotic chloramphenicol (Cm) and the opportunistic human pathogen *S*. *pneumoniae*. We show that Cm-resistant pneumococci expressing the resistance factor Cm acetyltransferase (CAT) can provide passive resistance for Cm-susceptible pneumococci by intracellular antibiotic deactivation. CAT covalently attaches an acetyl group from acetyl coenzyme A (acetyl-CoA) to Cm [26,27] and thus prevents the drug from binding to bacterial ribosomes [28]. Intracellular CAT in resistant bacteria can potently detoxify an entire environment in growth culture, semisolid surfaces of microscopy slides, or in a mouse infection model, supporting the survival and growth of genetically susceptible bacteria in the presence of initially effective Cm concentrations. Our results expand recent findings on the basis of *E*. *coli* growth cultures and indicate a potential clinical relevance of passive Cm resistance [29,30].

## Results

### Antibiotic Resistance of the Pneumococcus

Resistances to all currently prescribed antibiotics have been identified in clinical isolate strains of *S*. *pneumoniae* [31]. Genes that transfer antibiotic resistance can be classified according to their mode of action [32]. One class keeps the cytoplasmic drug level low by preventing drug entry or by exporting drug molecules. Another class alters the targeted enzymes by modifying their drug binding sites or by replacing the entire functional unit. A third class alters the drug molecules themselves. Only members of the latter group are potential candidates for establishing passive resistance. In the pneumococcus, resistance genes that deactivate antibiotics include aminoglycoside phosphor- or acetyltransferases and *cat*. To date, β-lactam antibiotic-degrading enzymes have not been reported in *S*. *pneumoniae* genomes or plasmids [33].

Standard therapy of pneumococcal infections does not include aminoglycosides because of the relatively high intrinsic resistance of *S*. *pneumoniae* to members of this antibiotic family. In contrast, Cm, a member of the World Health Organization Model List of Essential Medicines [34], is regularly prescribed throughout low-income countries for infections with *S*. *pneumoniae* and other Gram-positive pathogens due to its broad spectrum, oral availability, and excellent tissue distribution, including the central nervous system. Recently, the antibiotic was also discussed as candidate for a comeback in developed nations due to spreading resistances against first-line agents [35–37].

**Fig 1:**
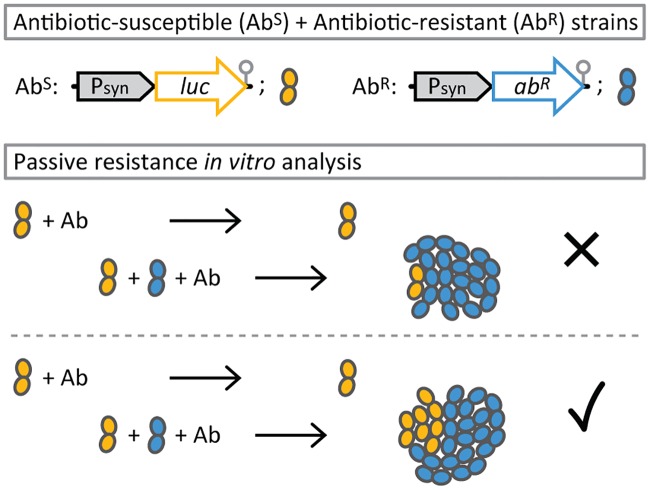
Experimental setup to determine passive resistance. Antibiotic-susceptible cells (Ab^S^) constitutively expressing *luc* are grown together with antibiotic-resistant cells (Ab^R^, which do not express *luc*). Only when the concentration of the antibiotic in the medium is reduced by enzymatic deactivation of resistant cells will the genetically antibiotic-susceptible cells be able to grow and produce light.

To test whether passive resistance emerges from antibiotic-deactivating resistance markers with *S*. *pneumoniae*, we used the drug-susceptible clinical isolate D39 [38]. We constructed an antibiotic-susceptible reporter strain expressing firefly luciferase (*luc*) and antibiotic-resistant strains expressing single-copy genomic integrated kanamycin 3′-phosphotransferase (*aphA1*), gentamicin 3′-acetyltransferase (*aacC1*), and chloramphenicol acetyltransferase (*cat*). Resistant and susceptible cells were grown at a one-to-one ratio, and optical density (both strains) and bioluminescence (emitted by susceptible cells only) were measured (Fig 1). Expression of *cat*, but not *aphA1* or *aacCI*, conferred passive resistance to susceptible cells (as observed by increased luminescence in mixed populations compared with assays of susceptible cells only; S1 Fig), mirroring prior investigations of antibiotic deactivation by resistant isolates of *S*. *pneumoniae* [39]. Aminoglycosides permeate the bacterial cell only at low frequency [40]; high permeability, however, was recently shown to represent an important precondition for the establishment of passive resistance, explaining why the phenomenon could not be observed with *aphA1* and *aacCI* expression [29].

### Collective Resistance to Cm In Vitro

**Fig 2:**
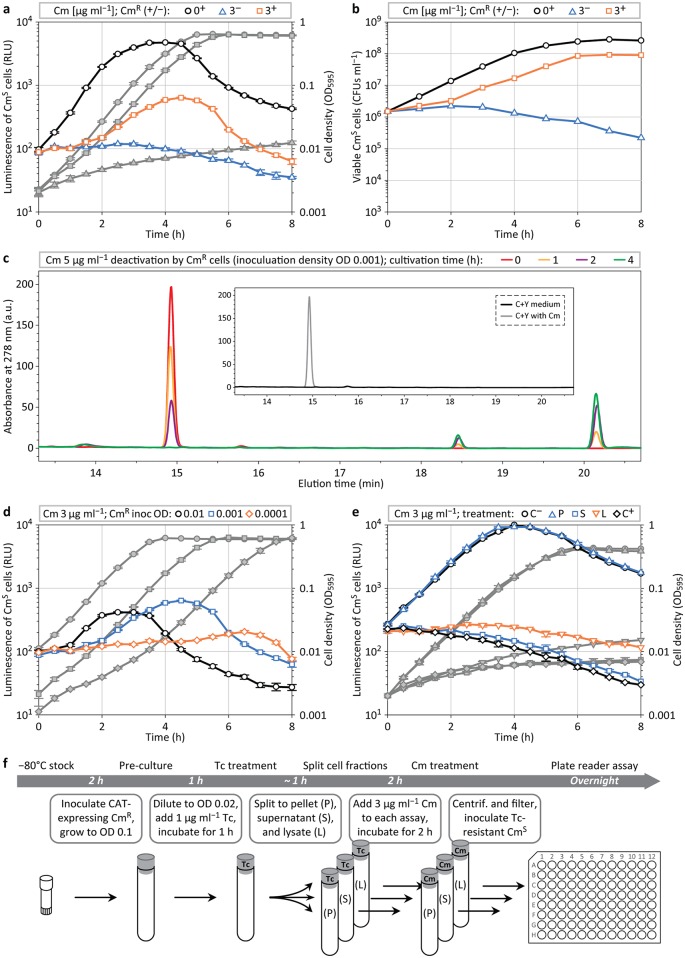
Cm deactivation during mixed population assays. (**a**) Plate reader assay sets in quadruplicate (average and standard error of the mean [s.e.m.]) measuring luminescence (symbols with color outline) and cell density (corresponding grey symbols) of *S*. *pneumoniae* Cm^S^ growing in the presence of 3 μg ml^−1^ Cm, in presence (+) or absence (−) of Cm^R^ cells. (**b**) Development of the count of viable Cm^S^ cells (colony-forming units per ml [CFUs ml^−1^]) during the cultivation assay presented in **a**, determined via plating in the presence of kanamycin; average values of duplicates are shown. (**c**) Culture supernatant (S) samples after 0, 1, 2, and 4 h of Cm^R^ cultivation (inoculation at optical density OD 0.001) in the presence of 5 μg ml^−1^ Cm, analyzed for Cm content by high-performance liquid chromatography (HPLC) separation and ultraviolet (UV) detection at 278 nm. (**d**) Luminescence and cell density profiles of Cm^S^ cells treated with 3 μg ml^−1^ Cm (inoculation at OD 0.001) in dependency of the inoculum size of Cm^R^ cells. (**e**, **f**), Cm^S^ luminescence and growth analysis (**e**) in Cm-supplemented medium (3 μg ml^−1^) that was pretreated with Cm^R^ cell pellet (P), S, and culture lysate (L), and controls without (C^−^) and with Cm (C^+^); (**f**) schematic overview of the assay (see also Methods and S1 Data).

To characterize the observed Cm collective resistance in more detail, we used the Cm-susceptible strain D-PEP2K1 (from here on Cm^S^), which constitutively expresses *luc* and the kanamycin resistance marker *aphA1* [41], and the Cm-resistant strain D-PEP1-pJS5 (from here on Cm^R^), which expresses *cat* from plasmid pJS5 [42] (see Methods). Luminescence allowed for the real-time estimation of growth (or inhibition) of the Cm^S^ population, and kanamycin resistance allowed for the monitoring of their viable cell count by plating assays in the presence of kanamycin. Cm represses the growth of susceptible pneumococci at a minimal inhibitory concentration (MIC) of 2.2 μg ml^−1^, and during Cm exposure, luminescence from *luc* expression of susceptible pneumococci was previously shown to decrease at a rate that depends on the applied Cm concentration [12]. However, when Cm^S^ was co-inoculated with CAT-expressing Cm^R^, luminescence (indicative for growth or inhibition of the Cm^S^ cell fraction) recovered, both for a Cm concentration slightly above the MIC (3 μg ml^−1^; Fig 2A) and even for a Cm concentration of more than two times the MIC (5 μg ml^−1^; S2 Fig). Luminescence recovery in mixed population assays (Cm^R^ + Cm^S^) exceeded the values measured with Cm^S^ monoculture by up to 10-fold (Fig 2A and S2 Fig), and plating assays (with kanamycin) revealed that the difference in viable cell count was 1,000-fold greater after 8 h of cocultivation (Fig 2B and S2 Fig). Although Cm is commonly regarded as bacteriostatic, bactericidal activity has also been demonstrated against *S*. *pneumoniae* [43], explaining the observed decrease in viability of Cm^S^ monoculture (Fig 2B and S2 Fig).

To confirm that Cm^R^ cells actually deactivate Cm in the growth medium, we analyzed culture supernatant (S) by high-performance liquid chromatography (HPLC) [44]. As shown in Fig 2C, within 4 h of growth, Cm^R^ cells entirely converted an initial Cm concentration of 5 μg ml^−1^, as evidenced by the disappearance of the corresponding Cm peak at wavelength 278 nm. New peaks (at later elution times) appeared and gradually increased in HPLC profiles of S collected after 1, 2, and 4 h of cultivation; these peaks were previously shown to correspond to acetylated Cm derivates (1- and 3-acetylchloramphenicol) [44].

Next, we focused on whether the initial amount of CAT-expressing Cm^R^ cells was important for the survival and growth of Cm^S^ cells during drug treatment. To test this, we inoculated microtiter plate wells with a fixed number of Cm^S^ cells (inoculation at optical density [OD] 0.001, corresponding to ~1.5 × 10^6^ colony-forming units per ml [CFUs ml^−1^]) while varying the number of Cm^R^ cells (Fig 2D). High inoculation densities of Cm^R^ cells (OD 0.01) resulted in a fast recovery of luminescence activity of Cm^S^ cells; however, the peak of luminescence was lower compared to intermediate Cm^R^ inoculation densities. This difference can be explained by cells reaching the carrying capacity of the growth medium before the pool of Cm is completely deactivated; luciferase expression activity was previously shown to slow down when cultures reach high cell densities (above ~OD 0.05) [41]. Relatively low Cm^R^ inoculation densities (OD 0.0001) also limited luminescence recovery of Cm^S^ cells during cocultivation. This finding likely reflects fewer Cm^R^ cells requiring more time to deactivate Cm, resulting in increased time spans of Cm^S^ drug exposure. Prolonged drug exposure of susceptible pneumococci was previously shown to result in increasing lag periods after drug removal, indicating a more severe perturbation of cell homeostasis [12]. The time span before outgrowth of Cm^S^ cells consequently consists of both the period required for drug clearance (by Cm^R^ cells) and the period required to reestablish intracellular conditions allowing for cell division.

### Intracellular Deactivation of Cm

To test whether Cm processing by CAT is an intracellular process, or if it takes place after secretion or cell lysis, we examined the potential of the S and the cytosolic content of Cm^R^ cells to deactivate Cm (assay scheme in Fig 2F). Precultured Cm^R^ cells were diluted to OD 0.02 and translation activity was blocked by adding 1 μg ml^−1^ tetracycline ([Tc]; *S*. *pneumoniae* D39 MIC: 0.26 μg ml^−1^) [12] for 1 h at 37°C to prevent ongoing protein synthesis and thus CAT expression. Next, the Tc-treated culture was split into three fractions: cell pellet (P) and S, separated via centrifugation, and cell culture lysate (L), obtained by sonication. The P was resuspended in C+Y medium containing 3 μg ml^−1^ Cm (and 1 μg ml^−1^ Tc), and 3 μg ml^−1^ Cm was added to the S and the L, followed by incubation at 37°C. After 2 h, the remaining cells and cell debris were removed by centrifugation and filtration, and the treated medium was used to test cell growth of a Tc-resistant variant of the Cm^S^ strain. Neither the S nor the L could support growth of Cm^S^, whereas medium preincubated with the P did (Fig 2E). Together, these experiments indicate that CAT is only active inside living cells, in which acetyl-CoA is present [26,27].

### Single-Cell Observations of Collective Resistance

Because the abovementioned experiments were performed in bulk assays, we wondered whether CAT-expressing bacteria would also efficiently deactivate Cm, and thus support the growth of susceptible cells, in a more complex environment, such as on semi-solid surfaces. To do so, we spotted Cm^R^ cells together with Cm-susceptible D-PEP33 cells expressing green fluorescent protein (GFP) on a matrix of 10% polyacrylamide C+Y medium containing 3 μg ml^−1^ Cm. Indeed Cm-susceptible D-PEP33 cells were able to grow and divide under these conditions (S3 Fig).

**Fig 3:**
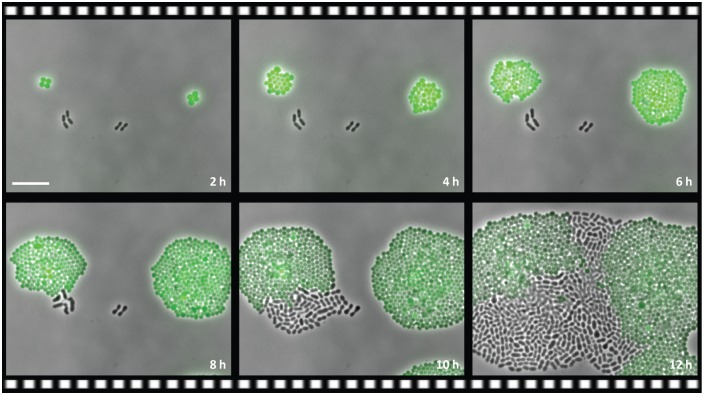
Interspecies collective resistance. Still images (overlay of phase contrast and fluorescence microscopy) of a time-lapse experiment of *S*. *pneumoniae* Cm^S^, cocultivated with a strain of the pneumococcal niche competitor *S*. *aureus* (strain LAC pCM29) that expresses CAT and GFP, growing on a semi-solid surface supplemented with 3 μg ml^−1^ Cm. Scale bar 10 μm.

*S*. *pneumoniae* cohabitates the human nasopharynx with other bacteria, such as *S*. *aureus* [6]. Therefore, we investigated whether CAT-expressing *S*. *aureus* could also support growth of Cm-susceptible *S*. *pneumoniae* in environments containing Cm. As shown in Fig 3 and S1 Movie, all *S*. *aureus* cells grew and divided from the starting point of the experiment, whereas *S*. *pneumoniae* Cm^S^ cells did not grow initially. However, after 8 h, a fraction of Cm^S^ cells grew out to form microcolonies. Note that Cm^S^ cells spotted in the absence of *S*. *aureus* did not grow under these conditions (S2 Movie).

### Requirements for Stable Coexistence

**Fig 4:**
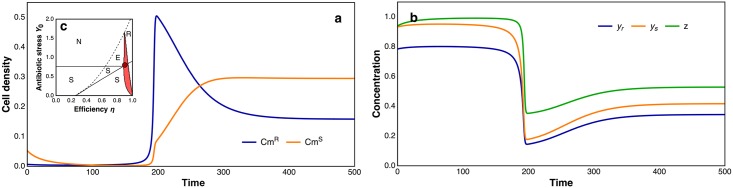
Population dynamics of bacterial communities. (**a**) Simulated growth trajectories for Cm^R^ and Cm^S^ populations subject to antibiotic stress and resource competition. (**b**) Dynamic of intracellular Cm (*y*_r_ and *y*_s_) and growth-limiting resource (*z*). Simulation time is scaled relative to the mean residence time of cells in a chemostat, which is equal to the generation time at steady state. At low population densities, the Cm^R^ strain can grow, whereas Cm^S^ cannot, due to a high concentration of Cm. However, the invasion of Cm^R^ lowers antibiotic stress, generating permissive conditions for the growth of Cm^S^ cells. The chemostat is then rapidly colonized by both strains (shortly after *t* = 180) until the resource becomes limiting. From that moment onwards, total cell density changes little, while the relative frequencies of the two strains continue to shift. Eventually, a stable equilibrium is reached, at which the cost and benefit of CAT expression (i.e., reduced growth rate efficiency for Cm^R^ cells versus their lower intracellular Cm concentration) balance out. Inset (**c**), The dark-red dot pinpoints the parameter set used in the simulation shown in **a** and **b**: *r* = 20.0, *η* = 0.9, *k*_z_ = 4.0, *c* = 1.0, *p* = 50.0, *h*_Y_ = 0.25/*Y*_0_, *k*_Y_ = 2.5/*Y*_0_, *d* = 30.0/*Y*_0_ and *Y*_0_ = 0.8. These parameters were selected to lie in a restricted area of parameter space (highlighted in red) where stable coexistence between Cm^S^ and Cm^R^ cells is observed Alternative model outcomes, which were identified by a numerical bifurcation analysis (see S1 Text and S4 Fig), include establishment of Cm^S^ only (area S), establishment of Cm^R^ only (area R), no bacterial growth (area N), and competition-induced extinction (area E, where Cm^S^ bacteria first outcompete Cm^R^ bacteria and subsequently are cleared by the antibiotic; see S5 Fig).

The observation that Cm^S^ cells grow only when Cm-deactivating cells are present in their close vicinity (Fig 3) suggests that the establishment of collective resistance requires Cm^S^ and Cm^R^ bacteria to be present in the same niche. However, such coexistence is subject to ecological constraints (e.g., the competitive exclusion principle) [45], particularly if susceptible and resistant strains compete for the same limiting resource. We therefore developed an ecological model to assess the scope for coexistence between CAT-producing bacteria and an antibiotic-susceptible strain (S1 Text). Consistent with this objective, we employed a minimalist modeling strategy and disentangled the qualitative effects of different factors (antibiotic stress, relative cost of Cm degradation and density regulation by ecological resource competition) from the interaction between Cm^S^ and Cm^R^ bacteria rather than aiming for a precise quantitative reconstruction of the experimental conditions. In fact, in contrast to natural environments (such as the human nasopharynx) that provide ample opportunities for coexistence because of spatial structure and concentration gradients of multiple resources, the model considers a worst-case scenario for coexistence: the two populations are assumed to grow in a well-mixed, homogeneous chemostat environment and are limited by the same resource. Nonetheless, we found that coexistence between Cm^R^ and Cm^S^ bacteria was feasible (Fig 4A and 4B), albeit under a restricted range of conditions (Fig 4C and S4 Fig). A mathematical analysis of the model (S1 Text) indicates that resistant and susceptible bacteria can establish a stable coexistence when CAT expression has a modest fitness cost. Without such a cost, the Cm^R^ strain is predicted to outcompete the Cm^S^ strain in the presence of antibiotics. Conversely, if the cost of expressing resistance is too high, the Cm^S^ strain will be the superior competitor. Interestingly, the model furthermore predicts parameter ranges that result in the extinction of mixed populations during drug treatment, while Cm^R^ populations on their own could survive (S4 and S5 Figs). A second condition for coexistence demands that the Cm^R^ population has a significant impact on the extracellular Cm concentration in its ecological niche. This requires that the population density reached at steady state must be high, so that coexistence can be stabilized by frequency-dependent selection, generated by a negative feedback loop between the relative abundance of drug-deactivating cells and the level of antibiotic stress in the environment.

We note that competitive exclusion acts at a local scale in structured environments, where the presence of spatial gradients in Cm and resources may help to create refuges in which either strain can escape competition from the other. In addition, we expect that coexistence between resistant and susceptible bacteria would be promoted in vivo by previously evolved ecological niche partitioning between co-occurring species.

### In Vivo Collective Cm Resistance

A general prediction from our mathematical model (S1 Text) is that coexistence of Cm^S^ and Cm^R^ in the presence of the antibiotic is precluded when the production of CAT carries no fitness cost; we expect this prediction to apply likewise in more complex environments with spatial and/or temporal heterogeneity in Cm concentrations. However, in vitro, in short-term experiments, we did not observe any obvious fitness cost for CAT expression (such as reduced growth rates or a reduced maximum cell density; Fig 2). Nevertheless, in vivo, a fitness cost might come into existence because resistant cells that grow rapidly might be preferentially targeted by the host innate immune system, as previously shown for commensal and pathogenic bacteria, including *E*. *coli* and *S*. *aureus* [46]. We tested the activity of the human antimicrobial peptide LL-37 in dependency of Cm treatment and found, indeed, increased killing efficiency against CAT-expressing *S*. *pneumoniae* (S6 Fig). Furthermore, although collective resistance could be successfully demonstrated in vitro, the phenomenon might not occur in more complex environments in vivo, such as in an animal infection model, because of a different flux balance between local Cm deactivation and restoration of effective drug concentrations via diffusion from surrounding tissues. To examine whether a coexistence between Cm^S^ and Cm^R^ is possible under therapy in vivo, we performed intratracheal infection of 8-wk-old female CD-1 mice with Cm^S^ alone and the combination of Cm^S^ + Cm^R^.

**Fig 5:**
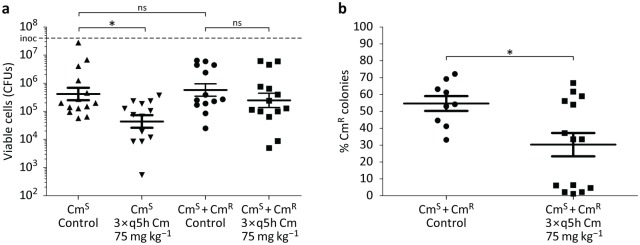
Cross-protection in a mouse pneumonia model. (**a**) Eight-wk-old female CD1 mice were infected intratracheally with Cm^S^ pneumococci or an equivalent amount of Cm^S^ + Cm^R^ pneumococci in a one-to-one ratio. One h post infection, mice were treated with one intraperitoneal injection of Cm 75 mg kg^−1^ followed by two additional doses spaced 5 h apart. Control mice received an injection of the vehicle alone. *n* = 14 for Cm^S^ control; 13 for Cm^S^ Cm-treated; 13 for Cm^S^ + Cm^R^ control; and 14 for Cm^S^ + Cm^R^ Cm-treated. Data plotted as average and s.e.m. of two independent experiments combined. Dashed line ‘inoc’ denotes the initial inoculum. *p < 0.*05*; one-way ANOVA with Tukey's multiple comparison post-test. (**b**) Bacterial colonies recovered from the Cm^S^ + Cm^R^ control and Cm^S^ + Cm^R^ Cm-treated mice were individually picked and used to inoculate THY media in 96-well plates. These 96-well plates were then used to inoculate 96-well plates with THY media containing either 15 μg ml^−1^ Cm or 100 μg ml^−1^ kanamycin to determine whether or not the original bacterial colony was Cm^S^ or Cm^R^. n = 9 for Cm^S^ + Cm^R^ control and 14 for Cm^S^ + Cm^R^ Cm-treated. Data plotted as average and s.e.m. of two independent experiments combined. *p = 0.04; Mann–Whitney *U* test (see S1 Data).

In the absence of Cm treatment, we observed no significant difference in the amount of viable bacteria recovered from the lungs 24 h after infection with Cm^S^ alone versus Cm^S^ + Cm^R^ at a one-to-one ratio (Fig 5A). When mice were given three doses of 75 mg kg^−1^ Cm once every 5 h, mice infected with Cm^S^ alone demonstrated a significant drop of one log-fold versus the untreated control. This is in contrast to mice coinfected with Cm^S^ + Cm^R^, in which Cm treatment did not significantly reduce the number of viable bacteria recovered from the lung versus the untreated control (Fig 5A). In the one-to-one mixed infection, approximately equal numbers of Cm^S^ and Cm^R^ cells were recovered in the absence of Cm treatment: 46% Cm^S^ and 54% Cm^R^ (Fig 5B). Surprisingly, with Cm treatment, 6 out of 14 animals had a dramatic increase in the percentage of Cm^S^ cells versus Cm^R^ cells. No pneumococcal colonies recovered could grow in both Cm- and kanamycin-containing media, excluding the possibility that horizontal gene transfer of the *cat* gene occurred during coinfection. Together, these results show that passive Cm resistance and the coexistence of resistant and susceptible cells also occur in vivo, associated with a fitness cost to the Cm^R^ niche members benefiting the Cm^S^ subpopulation.

## Discussion

This work elucidates that CAT, which is commonly found as a resistance marker in the human microbiome [47–49], can effectively protect Cm-susceptible pneumococci from the activity of the drug within local environments occupied by CAT-expressing cells. Because of its potency, long shelf life, and low cost, Cm remains a mainstay of broad-spectrum antibiotic therapy in several countries in Africa, the Indian subcontinent, and China [50]. The rise of multidrug resistance among human pathogens has also provoked interest in reevaluating Cm for certain serious infections in developed countries [35–37]. This work points out some caveats in using Cm to target human pathogens on mucosal surfaces because CAT-expressing commensals might provide passive resistance.

CAT can only deactivate Cm inside living cells (Fig 2E and 2F), presumably because it needs acetyl-CoA to acetylate and deactivate the target drug [26,27]. We show that Cm deactivation and collective resistance via CAT is not limited to *S*. *pneumoniae*, because CAT-expressing *S*. *aureus* can also support the local growth of pneumococci in the presence of initially effective Cm concentrations (Fig 3). Collective resistance by CAT does not only occur in vitro but also in vivo in a mouse pneumonia model (Fig 5). Strikingly, when Cm-treated mice were coinfected with CAT-expressing and Cm-susceptible pneumococci, the susceptible bacteria outcompeted the resistant ones (Fig 5). We previously showed that the susceptibility of bacteria towards antimicrobial peptides, produced by the host innate immune system, is markedly diminished in the presence of bacteriostatic antibiotics; Cm-inhibited *E*. *coli*, for example, are less efficiently cleared by the human peptide LL-37 [46]. We could demonstrate that this mechanism also takes place in *S*. *pneumoniae* (S6 Fig). When Cm was added to LL-37 treatment of pneumococci, the number of Cm^S^ cells recovered was one log-fold higher compared with Cm^R^ cells (S6 Fig). As shown before, this effect occurs because bacteriostatic antibiotics, such as Cm, inhibit the growth of susceptible bacteria and thereby reduce the susceptibility to host antimicrobial peptides that target bacterial division; Cm-resistant bacteria, in contrast, maintain fast growth in the presence of Cm and are therefore more rapidly killed by host antimicrobial peptides in vivo [46]. This phenomenon may therefore represent a contributing factor underlying our findings of the mouse pneumonia model. In this framework, rapidly growing Cm^R^ cells would suffer immune clearance, while the initially nongrowing Cm^S^ are less efficiently targeted by host defense factors. Once the Cm concentration has dropped sufficiently, Cm^S^ cells can outgrow and outcompete the diminished Cm^R^ population.

Our work with CAT and pneumococcus extends the known phenomenon of passive resistance via β-lactamase expression and expands on recent findings of collective resistance of bacterial communities [29,30]. Intracellular antibiotic deactivation requires a high drug permeability, and it is worth noting that this—in general desired—drug characteristic can also represent a risk factor for the effectiveness of an antibiotic therapy. Passive resistance could also appear with other antibiotic-degrading resistance factors in other bacteria [29] and may even emerge for synthetic antibiotic compounds [51]. In light of numerous reports of prevalence of drug resistance in pathogens, successful antibiotic therapy might become increasingly complicated with the occurrence of collective resistance. The phenomena could furthermore give rise to multidrug resistance of bacterial communities, in which individual resistances are expressed in different bacterial community residents [2,18,30]. Our mathematical model, however, predicts that collective resistance is only sustainable when resistance expression comes at a (modest) fitness cost (S4 Fig), and competitive exclusion is avoided by strong ecological feedback or alternative mechanisms (such as spatiotemporal structure or previously evolved niche partitioning). Nevertheless, even if coexistence is limited, the prolonged survival of susceptible cells within resistant communities may already represent an issue by increasing the opportunity for horizontal gene transfer during antibiotic selection pressure. Passive resistance might consequently represent an important factor towards the development of genetic multidrug resistance.

## Methods

### Strains and Growth Conditions

*S*. *pneumoniae* Cm^S^, a Cm-susceptible D39 derivate strain that constitutively expresses *luc* and a kanamycin resistance marker was used throughout. The Tc-resistant variant of this strain contained the Tc resistance gene *tetM* integrated at the *bgaA* locus, obtained via transformation with pPP1 [52]. *luc* has a reported half-life of 3 min in *S*. *pneumoniae*, and luminescence therefore gives real-time information on the level of gene expression activity [41,53]. *S*. *pneumoniae* Cm^R^, expressing CAT from plasmid pJS5, was used as standard for a Cm-resistant strain. Initial experiments were carried out with the Cm-resistant strain D-PEP1C3 that expresses CAT from a strong synthetic promoter. *S*. *pneumoniae* D-PEP33 expressing GFP was used as a Cm-susceptible strain in time-lapse microscopy experiments [41]. *S*. *aureus* experiments were performed with strain LAC pCM29 [54] that constitutively expresses CAT and GFP.

*S*. *pneumoniae* and *S*. *aureus* cells were grown in C+Y medium (pH 6.8), supplemented with 0.5 μg ml^−1^ D-luciferin for luminescence measurements, at 37°C [55]. Pre-cultures for all experiments were obtained by a standardized protocol, in which previously exponentially growing cells from −80°C stocks were diluted to OD (600 nm, path length 10 mm) 0.005 and grown until OD 0.1 in a volume of 2 ml medium inside tubes that allow for direct in-tube OD measurements. To determine the number of colony-forming units (CFUs), *S*. *pneumoniae* cells were plated inside Columbia agar supplemented with 3% (v v^−1^) sheep blood and incubated overnight at 37°C.

### Microtiter Plate Reader Assays

Costar 96-well plates (white, clear bottom) with a total assay volume of 300 μl per well were inoculated to the designated starting OD value. Microtiter plate reader experiments were performed using a TECAN infinite pro 200 plate reader (Tecan Group) by measuring every 10 min with the following protocol: 5 s shaking, OD (595 nm, path length 10 mm) measurement with 25 flashes, luminescence measurement with an integration time of 1 s.

In mixed population assays (shown in Fig 2A), all cultures were inoculated with Cm^S^ cells to an initial cell density of OD 0.001. Cm^R^ cells were inoculated to the same density, and control cultures without Cm^R^ cells contained equal amounts of Cm-sensitive D39 wild-type cells to correct for unspecific effects such as drug-titration via cellular Cm binding.

### HPLC Analysis

S were obtained by Cm^R^ cultivation (inoculation at OD 0.001) in the presence of 5 μg ml^−1^ Cm in microtiter plates (as described above). Four wells were sampled and pooled per time point (combined volume of 1.2 ml), centrifuged to remove cells, and filtered through a 0.2 μm filter. HPLC analysis was carried out using an Agilent 1260 Infinity system (Agilent Technologies) with ultraviolet (UV) detection at 278 nm (maximum absorbance of Cm) [44]. An Aeris Peptide XB-C18 column (Phenomenex) with 3.6 μm particle and a size of 250 × 4.60 mm was used. Reversed-phase chromatography was carried out at a constant flow rate of 1 ml min^−1^, with the mobile phase consisting of solution A: 10 mM sodium acetate buffer (pH 6.0) containing 5% acetonitrile (v v^−1^) and solution B: acetonitrile 0.1% TFA, according to the following protocol: 100 μl sample loading, 3 min 10% B, 20 min gradient 10% to 50% B, 1 min gradient 50% to 95% B, 3 min 95% B, 1 min gradient 95% to 10% B, 6 min 10% B.

### Microscopy

A Nikon Ti-E microscope equipped with a CoolsnapHQ2 camera and an Intensilight light source was used. Time-lapse microscopy was carried out by spotting pre-cultured cells on 10% polyacrylamide slides inside a Gene Frame (Thermo Fisher Scientific) that was sealed with the cover glass to guarantee stable conditions during microscopy. The polyacrylamide slide was prepared with growth medium containing 3 μg ml^−1^ Cm. Images of fluorescing cells were taken with the following protocol and filter settings: 0.3 s exposure for phase contrast, 0.5 s exposure for fluorescence at 440–490 nm excitation via a dichroic mirror of 495 nm, and an emission filter of 500–550 nm. Temperature during microscopy was controlled by an Okolab climate incubator, and images were taken every 10 min during 20 h at 37°C.

### Mouse Infection Model

The murine pneumonia model was performed with slight modifications as previously described [56]. Based on pilot experiments, we estimated that the number of animals required to observe a statistical difference between the groups would exceed the technical limit of animals that could be inoculated and treated per day. Therefore, the experiment was split into 2 d with the original pool of animals randomized to each group at the start of the multi-day experiment. Prior to statistical analysis, the data were combined. Note that all intratracheal infections were performed in a blinded fashion with respect to Cm or vehicle treatment. Eight-wk-old female CD1 mice (Charles River Laboratories) with an average body weight of 28 g were used. Fresh cultures of Cm^S^ and Cm^R^ were started in 10 ml of Todd-Hewitt broth containing 2% yeast extract (THY) and 10 ml of THY supplemented with 5 μg ml^−1^ Cm, respectively. Cultures were grown at 37°C in a 5% CO_2_ incubator until OD (600 nm) 0.6. Bacteria were washed twice with PBS via centrifugation at 3,220 × g at room temperature and concentrated in PBS to yield 3.5 × 10^7^ CFU in the inoculation volume of 40 μl. For mixed infections, an equal volume of concentrated Cm^S^ and Cm^R^ pneumococci were combined. Mice were anesthetized with 100 mg kg^−1^ ketamine and 10 mg kg^−1^ xylazine. Once sedated, the vocal chords were visualized using an operating otoscope (Welch Allyn), and 40 μl of bacteria was instilled into the trachea during inspiration using a plastic gel loading pipette tip. Mice were placed on a warmed pad for recovery. After 1 h, one intraperitoneal injection of Cm 75 mg kg^−1^ or vehicle controls was given, followed by two additional doses spaced 5 h apart. Mice were sacrificed with CO_2_ 24 h after infection.

To enumerate total surviving bacteria in the lungs, both lung lobes were removed and placed in a 2 ml sterile micro tube (Sarstedt) containing 1 ml of PBS and 1 mm silica beads (Biospec). Lungs were homogenized by shaking twice at 6,000 rpm for 1 min using a MagNA Lyser (Roche), with the specimens placed on ice as soon as they were harvested. Aliquots from each tube were serially diluted for CFU enumeration on THY plates. To determine whether or not a colony was Cm^S^ or Cm^R^, individual colonies from the THY plates were picked and transferred into 100 μl of THY media in 96-well plates. The 96-well plates were incubated overnight at 37°C in a 5% CO_2_ incubator. After overnight incubation, wells were mixed, and 5 μl of media from each well was transferred into 100 μl of THY containing 15 μg ml^−1^ Cm or 100 μg ml^−1^ kanamycin. The 96-well plates were once again incubated overnight at 37°C in a 5% CO_2_ incubator, and wells were finally assessed for the presence or absence of a bacterial P. Cm (≥98% purity; Sigma) for animal injection was prepared as follows: 40 mg ml^−1^ of Cm was dissolved in 800 μl of 70% ethanol in PBS to make a 50 mg ml^−1^ stock solution. This stock solution was diluted in PBS to 3.75 mg ml^−1^ for intraperitoneal injection into mice at 75 mg kg^−1^.

### Ethics Statement

This study was carried out in strict accordance with the recommendations in the Guide for the Care and Use of Laboratory Animals of the National Institutes of Health. The corresponding protocol entitled “Mouse Models of Bacterial Infection and Innate Immunity” (#S00227M) was approved by the Institutional Animal Care and Use Committee of the University of California, San Diego (Animal Welfare Assurance Number: A3033-01). All efforts were made to minimize suffering of animals employed in this study.

### Modeling

The model describes the dynamic of a coculture of CAT-expressing Cm^R^ and Cm^S^ bacterial cells growing in the presence of Cm in a chemostat environment. The two strains, with population densities *x*_r_ and *x*_s_, respectively, compete for a growth-limiting resource, *z*. Cm is assumed to inhibit growth; we separately keep track of the intracellular concentrations of Cm (*y*_s_ in susceptible cells and *y*_r_ in resistant cells) and its concentration in the extracellular medium *y*_m_. The equations for the growth of the two bacterial populations and the growth-limiting resource are given by

$$\begin{array}{ccc} \frac{dx_{\text{s}}}{d\tau } & = & r\frac{z}{k_{\text{z}}+z}\frac{h_{\text{y}}}{h_{\text{y}}+y_{\text{s}}}x_{\text{s}}-x_{\text{s}},\\ \frac{dx_{\text{r}}}{d\tau } & = & \eta r\frac{z}{k_{\text{z}}+z}\frac{h_{\text{y}}}{h_{\text{y}}+y_{\text{r}}}x_{\text{r}}-x_{\text{r}},\\ \frac{dz}{d\tau } & = & \left(1-z\right)-cr\frac{z}{k_{\text{z}}+z}\left(x_{\text{s}}\frac{h_{\text{y}}}{h_{\text{y}}+y_{\text{s}}}-x_{\text{r}}\frac{h_{\text{y}}}{h_{\text{y}}+y_{\text{r}}}\right), \end{array}$$ (1)

where *r* is the maximum growth rate of Cm^S^ cells, *η* is the relative growth efficiency of Cm^R^ cells, *c* is the resource consumption rate, and *k*_z_ and *h*_y_, respectively, are the half-saturation and inhibitory constants of the growth function. Time, resource concentration, and cell densities have been scaled relative to the flow rate of the chemostat, the resource concentration in the inflow medium, and the number of cells that fit in the chemostat volume, respectively, in order to reduce the number of free parameters (see S1 Text for details).

The concentrations of Cm in the different compartments, which have been scaled relative to the Cm concentration in the inflow medium, change according to the equations:

$$\begin{array}{ccc} \frac{dy_{\text{m}}}{d\tau } & = & \frac{1}{1-x_{\text{s}}-x_{\text{r}}}-p\frac{x_{\text{s}}\left(y_{\text{m}}-y_{\text{s}}\right)+x_{\text{r}}\left(y_{\text{m}}-y_{\text{r}}\right)}{1-x_{\text{s}}-x_{\text{r}}}-y_{\text{m}}-y_{\text{m}}\frac{d\text{ln}\left(1-x_{\text{s}}-x_{\text{r}}\right)}{d\tau },\\ \frac{dy_{\text{s}}}{d\tau } & = & p\left(y_{\text{m}}-y_{\text{s}}\right)-y_{\text{s}}-y_{\text{s}}\frac{d\text{ln}x_{\text{s}}}{d\tau },\\ \frac{dy_{\text{r}}}{d\tau } & = & p\left(y_{\text{m}}-y_{\text{r}}\right)-d\frac{y_{\text{r}}}{k_{\text{y}}+y_{\text{r}}}-y_{\text{r}}-y_{\text{r}}\frac{d\text{ln}x_{\text{r}}}{d\tau }. \end{array}$$ (2)

The processes described by the terms on the right-hand side include inflow of Cm into the medium, passive transport of Cm between compartments at rate *p*, outflow from the chemostat, degradation of Cm by CAT in Cm^R^ cells (according to Michaelis–Menten kinetics with maximum rate *d* and half-saturation constant *k*_y_), and concentration changes due to fluctuations in the volume of the compartments. Eqs (1) and (2) were solved numerically using *Mathematica* (Wolfram) or simulation software written in C^++^ (used for the numerical bifurcation analysis, based on a Runge–Kutta integration algorithm with adaptive step-size control).

## Supporting Information

S1 Fig Antibiotic degradation in the pneumococcus. (**a**–**c**), Plate reader assay sets in quadruplicate (average and s.e.m.) measuring luminescence (symbols with color outline) and cell density (corresponding grey symbols) of antibiotic-resistant (Ab^R^), antibiotic-susceptible (Ab^S^) and a mixture of resistant and susceptible (Ab^R^+Ab^S^) *S*. *pneumoniae* cells growing in the presence of 200 μg ml^−1^ kanamycin (**a**), 20 μg ml^−1^ gentamycin (**b**), and 3 μg ml^−1^ chloramphenicol (**c**). Assays with resistant cells (Ab^R^) were inoculated to a density of OD 0.002, mixed populations (Ab^R^+Ab^S^) to a density of OD 0.001 each, and susceptible cells-only (Ab^S^) also to a density of OD 0.001 with the addition of equal amounts of D39 wild type cells to correct for unspecific effects such as cellular drug binding. D-PEP22 that constitutively expresses firefly luciferase was used throughout as susceptible strain. Resistant strains expressed *aphA1* (**a**), *aacCI* (**b**), and *cat*(**c**). Note that in aminoglycoside-inhibited cultures (**a** and **b**) luminescence of Ab^R^+Ab^S^ assays decreased more rapidly compared with Ab^S^ assays. This can be explained by reduced luciferase expression rates when cultures exceed OD 0.05; Ab^S^cultures, in contrast to Ab^R^+Ab^S^ cultures, do not reach OD 0.05 and consequently continue to express luciferase at a higher rate [41] (see S1 Data).
(TIF)

S2 Fig Chloramphenicol deactivation assay at a concentration of two times the MIC. (**a**), Plate reader assay sets in quadruplicate (average and s.e.m.) measuring luminescence (symbols with color outline) and cell density (corresponding grey symbols) of chloramphenicol-susceptible *S*. *pneumoniae* D-PEP2K1 (Cm^S^) growing in the presence of 5 μg ml^−1^ chloramphenicol (Cm), in presence (+) or absence (−) of resistant D-PEP1-pJS5 (Cm^R^) cells. (**b**), Development of the count of viable Cm^S^ cells (CFUs ml^−1^, colony-forming units per ml) during the cultivation assay presented in **a**, determined via plating in the presence of kanamycin; average values of duplicates are shown (see S1 Data).
(TIF)

S3 Fig Single-cell analysis of pneumococcal collective resistance. (**a**,**b**), Still images (overlay of phase contrast and fluorescence microscopy) of a time-lapse experiment of chloramphenicol-susceptible *S*. *pneumoniae* D-PEP33 cells that constitutively express GFP, either co-cultivated with the CAT-expressing *S*. *pneumoniae* D-PEP1-pJS5 (**a**) or in monoculture (**b**), growing on a semi-solid surface supplemented with 3 μg ml^−1^ chloramphenicol. High inoculation densities were spotted, resulting in rapid chloramphenicol deactivation in the co-cultivation assay. Note that GFP, which allows for the distinction between chloramphenicol-susceptible and -resistant cells at the beginning of the time-lapse experiment (fluorescent versus non-fluorescent), bleaches quickly in the course of the assay; inhibited susceptible cells, even after (partial) Cm clearance, do not express sufficient levels of GFP (counteracting photobleaching) to allow for a continuous detection. Scale bar, 10 μm.
(TIF)

S4 Fig Numerical model analysis. (**a**–**c**), Colored areas indicate qualitatively different outcomes of competition between Cm^S^ and Cm^R^ cells in model simulations, as a function of two key parameters: the growth rate efficiency of Cm^R^ cells (1 – *η* quantifies the cost of CAT expression), and the concentration of Cm in the inflow medium (*Y*_0_; ‘antibiotic stress’). (**a**), An orange line borders the region in which the Cm^S^ strain can grow from low initial density. This is below a critical level of antibiotic stress, or in a narrow range of *η* values when the resistant cells are present. The Cm^R^ strain can grow from low initial density in the area bordered by a solid blue line, but can maintain high population densities over a larger area of parameter space (i.e., in the area bordered by a dashed blue line). Stable coexistence of both strains is maintained in a narrow parameter region (red area). When *η* is close to 1, Cm^R^ is always a superior competitor (dark blue area), in line with the analytical result that coexistence cannot be maintained unless CAT expression is costly. When CAT expression costs are high, Cm^S^ tends to outcompete Cm^R^. However, this process leads to an elevation of Cm in the medium, which may eventually cause both strains to go extinct (light blue area; here, Cm^R^ can survive on its own, but not when Cm^S^ is also present; see S5 Fig). Alternatively, Cm^S^ can persist on its own after driving Cm^R^ to extinction (orange area). Parameters are: *r* = 20.0, *k*_z_ = 4.0, *c* = 1.0, *p* = 50.0, *h*_Y_ = 0.25/*Y*_0_, *k*_Y_ = 2.5/*Y*_0_ and *d* = 30.0/*Y*_0_. In (**b**), the relative benefit of CAT degradation is larger, due to a slower diffusion of Cm across the cell membrane (*p* = 25.0; other parameters as in **a**). (**c**), This panel illustrates the effect of a change in the resource consumption rate *c* which affects the equilibrium population densities (*c* = 2.0; other parameters as in **a**). In this case, Cm^R^ and Cm^S^ reach lower equilibrium densities, weakening the effect of Cm^R^ on the environment. As a result, the conditions for coexistence become more stringent. Throughout, we performed multiple simulations per parameter condition to search for boundary and interior equilibria, and classified the dynamics based on the stability properties of the equilibria. Color saturation within each area gives an indication of the total cell density at equilibrium.
(TIF)

S5 Fig Extinction induced by competition. (**a**), Simulated growth trajectories for Cm^R^ and Cm^S^ populations subject to antibiotic stress and competition for a limiting resource. Here, the Cm^R^ strain is an inferior competitor that is driven to extinction by the invasion of Cm^S^ cells, even though the growth conditions are not permissive for the survival of Cm^S^ on its own. Extinction is caused by a bistability in the growth dynamic of Cm^R^ cells: a critical cell density is required to lower the concentration of Cm below the level that permits population growth. The initial Cm^R^ cell density in the simulation was just above this critical level (indicated by a dotted gray line); the Cm^R^ cells are not able to invade if their initial density lies below the threshold (shown by the dashed blue trajectory). However, after successful invasion (solid blue trajectory), the Cm^R^ cells can still be pushed below their critical density by competition with the Cm^S^ strain, triggering the collapse of both populations. (**b**), Dynamics of intracellular Cm concentrations and resource. Parameters are: *r* = 20.0, *η* = 0.85, *k*_z_ = 4.0, *c* = 1.0, *p* = 50.0, *h*^Y^ = 0.25, *k*_Y_ = 2.5 and *d* = 30.0.
(TIF)

S6 Fig LL-37 activity in dependency on chloramphenicol. Killing of Cm-susceptible *S*. *neumoniae* D-PEP2K1 (Cm^S^) and Cm-resistant D-PEP1C3 (Cm^R^) by the human antimicrobial peptide LL-37 at a concentration of 50 μg ml^−1^, in absence (−) or presence (+) of 5 μg ml^−1^ chloramphenicol (Cm); average and s.e.m. of duplicates are shown. *P< 0.05; two-tailed *t*-test (see S1 Data).
(TIF)

S1 Text Derivation of the mathematical model and model analysis. (PDF)

S1 Data Numerical values underlying the data presented in the figures. (XLSX)

S1 Movie Development of interspecies collective resistance. Time-lapse microscopy experiment of *S*. *pneumoniae* D-PEP2K1 (Cm^S^), co-cultivated with a strain of the pneumococcal niche competitor *Staphylococcus aureus* that expresses CAT and GFP (strain LAC pCM29), growing on a semi-solid surface containing 3 μg ml^−1^ chloramphenicol. The first still frame of the time-lapse experiment is annotated as one hour into the cultivation start (01:00); one hour was the time required for reaching stable conditions inside the microscopy slide that allow for automated recording. Note that GFP expression, in the case of the Cm-resistant *S*. *aureus* LAC pCM29, is not inhibited by the Cm treatment, and GFP is consequently continuously produced (counteracting photobleaching and dilution). The observed high fluorescence is the result of GFP expression from a multi-copy plasmid (in contrast to the single-copy genomic integration in *S*. *pneumoniae* D-PEP33 shown in S3 Fig).
(MP4)

S2 Movie Chloramphenicol-treated susceptible pneumococci. Time-lapse microscopy experiment of chloramphenicol-susceptible *S*. *pneumoniae* D-PEP2K1 (Cm^S^) monoculture growing on a semi-solid surface containing 3 μg ml^−1^ chloramphenicol.
(MP4)

## Abbreviations

*aacC1*: gentamicin 3′-acetyltransferase
acetyl-CoA: acetyl coenzyme A
*aphA1*: kanamycin 3′-phosphotransferase
CAT: chloramphenicol acetyltransferase
CFU: colony-forming unit
CFUs ml^−1^: colony-forming units per ml
Cm: chloramphenicol
Cm^R^: Cm-resistant strain D-PEP1-pJS5
Cm^S^: CM-susceptible strain D-PEP2K1
GFP: green fluorescent protein
HPLC: high-performance liquid chromatography
L: lysate
*luc*: firefly luciferase
MIC: minimal inhibitory concentration
OD: optical density
P: cell pellet
S: culture supernatant
s.e.m.: standard error of the mean
Tc: tetracycline
UV: ultraviolet
