# Supplementary figures and images for: Collective Resistance in Microbial Communities by Intracellular Antibiotic Deactivation
Source: PLoS Biol. 2016 Dec 27;14(12):e2000631. doi: 10.1371/journal.pbio.2000631 (PMC5189934; doi:10.1371/journal.pbio.2000631)

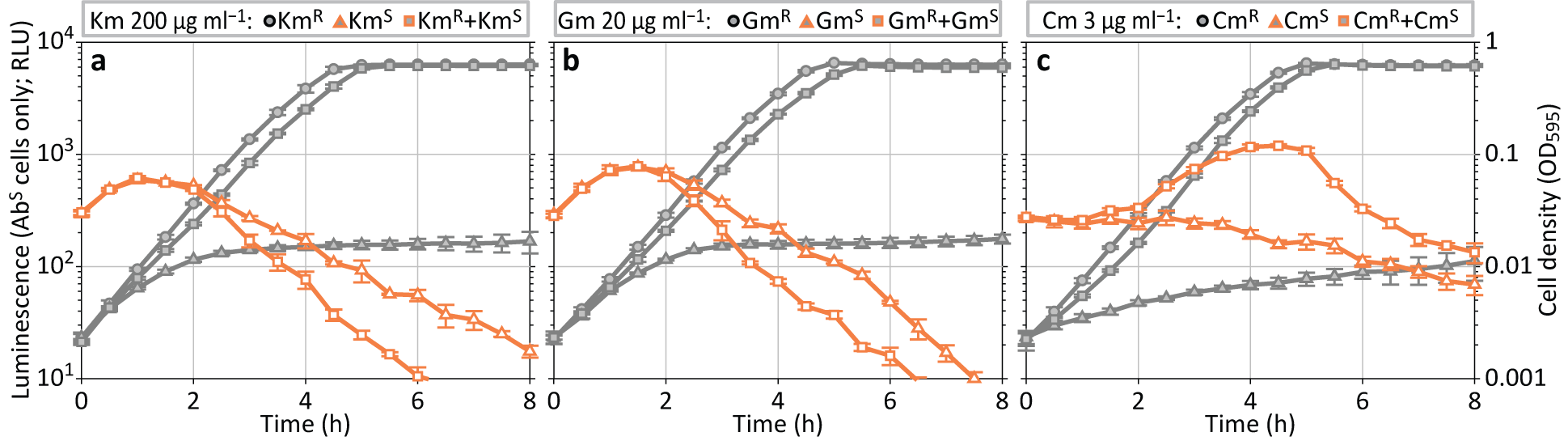

Supplement: S1 Fig — (a–c), Plate reader assay sets in quadruplicate (average and s.e.m.) measuring luminescence (symbols with color outline) and cell density (corresponding grey symbols) of antibiotic-resistant (AbR), antibiotic-susceptible (AbS) and a mixture of resistant and susceptible (AbR+AbS) S. pneumoniae cells growing in the presence of 200 μg ml−1 kanamycin (a), 20 μg ml−1 gentamycin (b), and 3 μg ml−1 chloramphenicol (c). Assays with resistant cells (AbR) were inoculated to a density of OD 0.002, mixed populations (AbR+AbS) to a density of OD 0.001 each, and susceptible cells-only (AbS) also to a density of OD 0.001 with the addition of equal amounts of D39 wild type cells to correct for unspecific effects such as cellular drug binding. D-PEP22 that constitutively expresses firefly luciferase was used throughout as susceptible strain. Resistant strains expressed aphA1 (a), aacCI (b), and cat(c). Note that in aminoglycoside-inhibited cultures (a and b) luminescence of AbR+AbS assays decreased more rapidly compared with AbS assays. This can be explained by reduced luciferase expression rates when cultures exceed OD 0.05; AbScultures, in contrast to AbR+AbS cultures, do not reach OD 0.05 and consequently continue to express luciferase at a higher rate [41] (see S1 Data). (TIF) [file pbio.2000631.s001.tif]

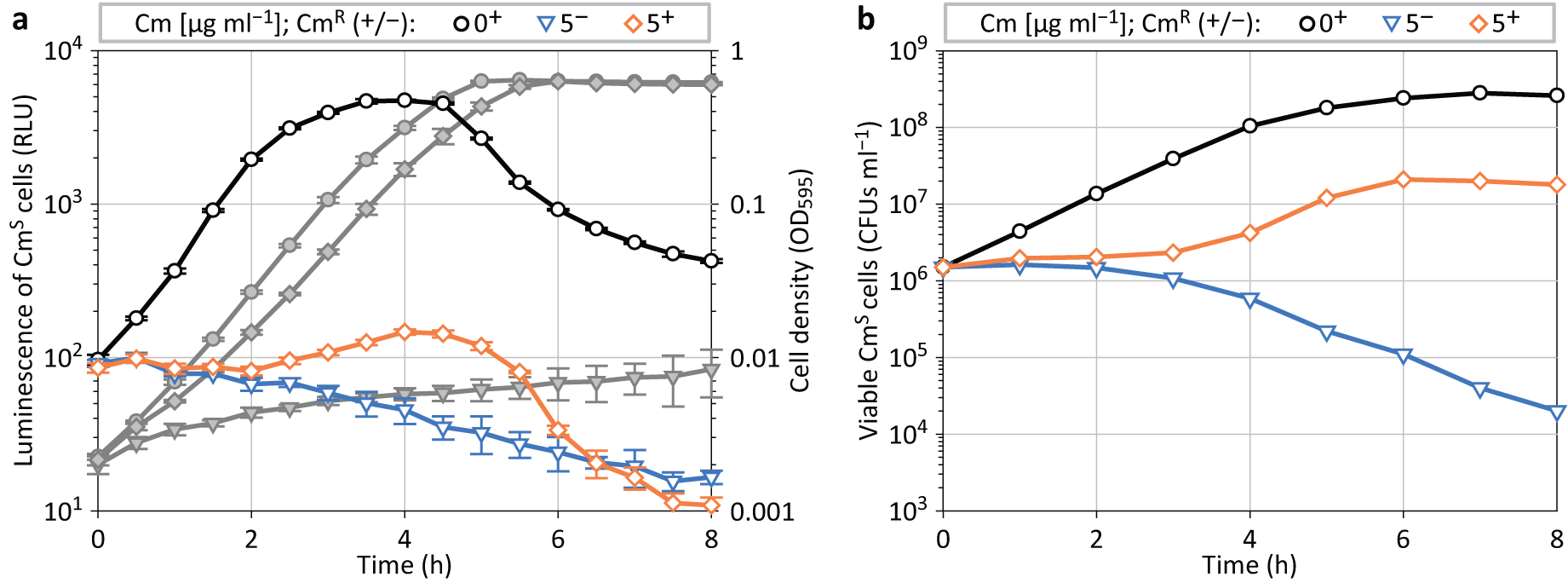

Supplement: S2 Fig — (a), Plate reader assay sets in quadruplicate (average and s.e.m.) measuring luminescence (symbols with color outline) and cell density (corresponding grey symbols) of chloramphenicol-susceptible S. pneumoniae D-PEP2K1 (CmS) growing in the presence of 5 μg ml−1 chloramphenicol (Cm), in presence (+) or absence (−) of resistant D-PEP1-pJS5 (CmR) cells. (b), Development of the count of viable CmS cells (CFUs ml−1, colony-forming units per ml) during the cultivation assay presented in a, determined via plating in the presence of kanamycin; average values of duplicates are shown (see S1 Data). (TIF) [file pbio.2000631.s002.tif]

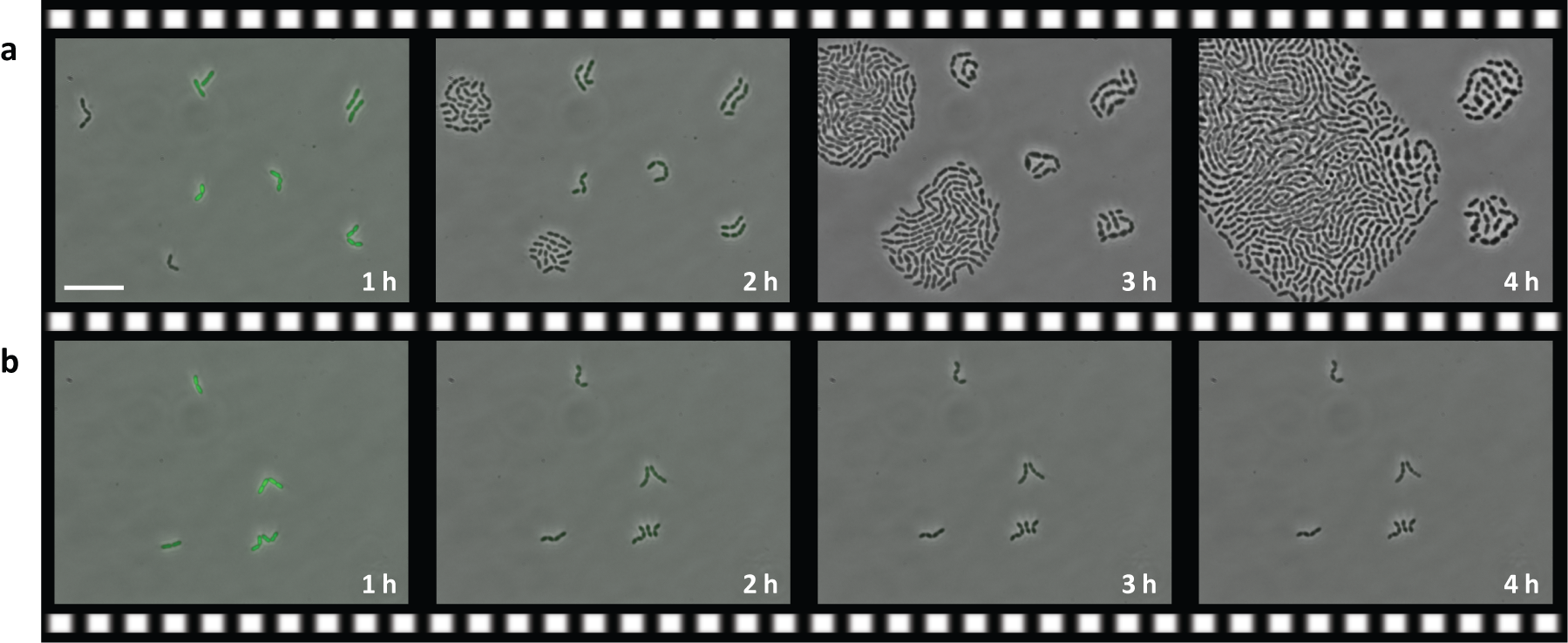

Supplement: S3 Fig — (a,b), Still images (overlay of phase contrast and fluorescence microscopy) of a time-lapse experiment of chloramphenicol-susceptible S. pneumoniae D-PEP33 cells that constitutively express GFP, either co-cultivated with the CAT-expressing S. pneumoniae D-PEP1-pJS5 (a) or in monoculture (b), growing on a semi-solid surface supplemented with 3 μg ml−1 chloramphenicol. High inoculation densities were spotted, resulting in rapid chloramphenicol deactivation in the co-cultivation assay. Note that GFP, which allows for the distinction between chloramphenicol-susceptible and -resistant cells at the beginning of the time-lapse experiment (fluorescent versus non-fluorescent), bleaches quickly in the course of the assay; inhibited susceptible cells, even after (partial) Cm clearance, do not express sufficient levels of GFP (counteracting photobleaching) to allow for a continuous detection. Scale bar, 10 μm. (TIF) [file pbio.2000631.s003.tif]

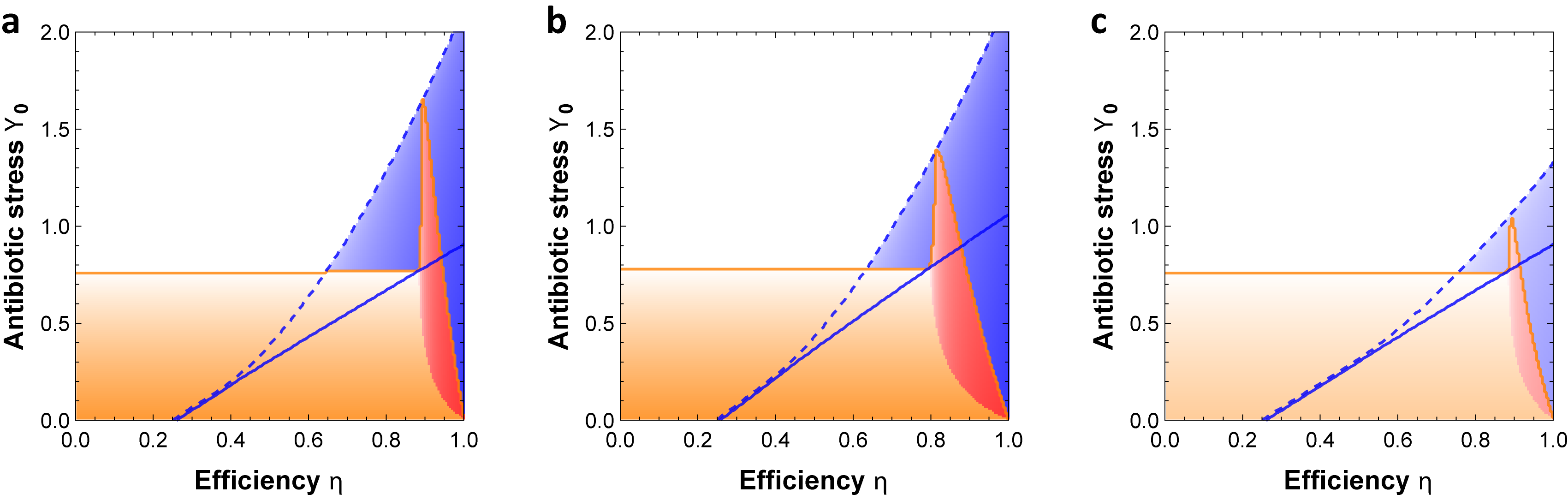

Supplement: S4 Fig — (a–c), Colored areas indicate qualitatively different outcomes of competition between CmS and CmR cells in model simulations, as a function of two key parameters: the growth rate efficiency of CmR cells (1 – η quantifies the cost of CAT expression), and the concentration of Cm in the inflow medium (Y0; ‘antibiotic stress’). (a), An orange line borders the region in which the CmS strain can grow from low initial density. This is below a critical level of antibiotic stress, or in a narrow range of η values when the resistant cells are present. The CmR strain can grow from low initial density in the area bordered by a solid blue line, but can maintain high population densities over a larger area of parameter space (i.e., in the area bordered by a dashed blue line). Stable coexistence of both strains is maintained in a narrow parameter region (red area). When η is close to 1, CmR is always a superior competitor (dark blue area), in line with the analytical result that coexistence cannot be maintained unless CAT expression is costly. When CAT expression costs are high, CmS tends to outcompete CmR. However, this process leads to an elevation of Cm in the medium, which may eventually cause both strains to go extinct (light blue area; here, CmR can survive on its own, but not when CmS is also present; see S5 Fig). Alternatively, CmS can persist on its own after driving CmR to extinction (orange area). Parameters are: r = 20.0, kz = 4.0, c = 1.0, p = 50.0, hY = 0.25/Y0, kY = 2.5/Y0 and d = 30.0/Y0. In (b), the relative benefit of CAT degradation is larger, due to a slower diffusion of Cm across the cell membrane (p = 25.0; other parameters as in a). (c), This panel illustrates the effect of a change in the resource consumption rate c which affects the equilibrium population densities (c = 2.0; other parameters as in a). In this case, CmR and CmS reach lower equilibrium densities, weakening the effect of CmR on the environment. As a result, the conditions for coexistence beco [file pbio.2000631.s004.tif]

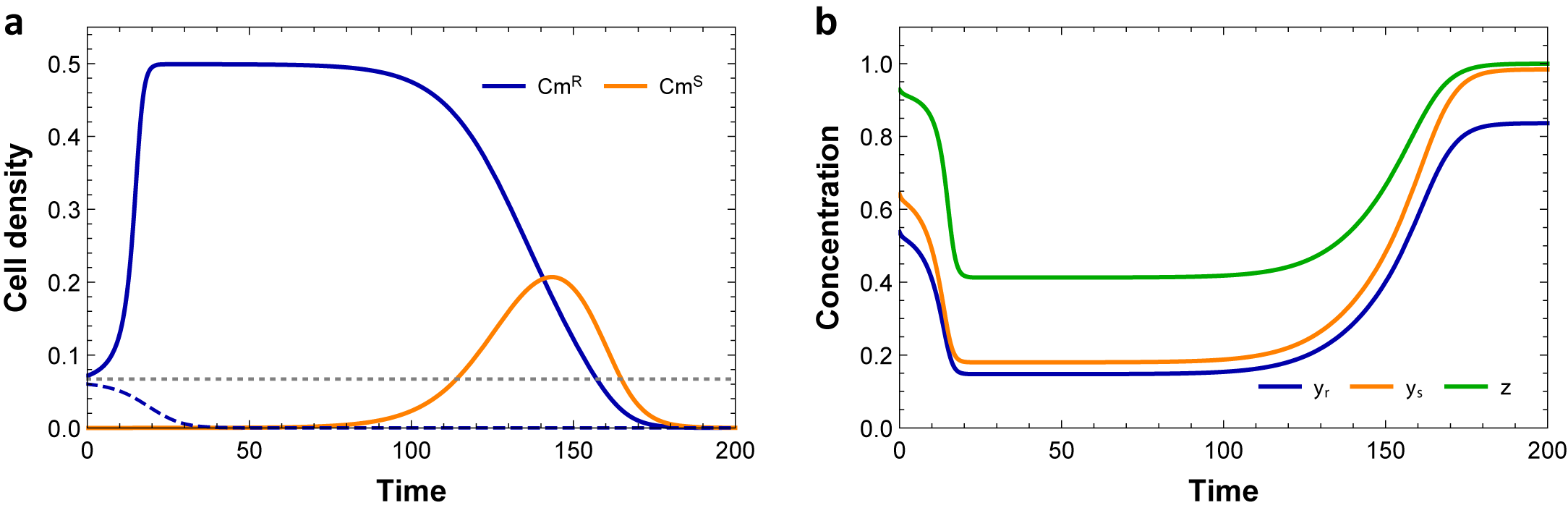

Supplement: S5 Fig — (a), Simulated growth trajectories for CmR and CmS populations subject to antibiotic stress and competition for a limiting resource. Here, the CmR strain is an inferior competitor that is driven to extinction by the invasion of CmS cells, even though the growth conditions are not permissive for the survival of CmS on its own. Extinction is caused by a bistability in the growth dynamic of CmR cells: a critical cell density is required to lower the concentration of Cm below the level that permits population growth. The initial CmR cell density in the simulation was just above this critical level (indicated by a dotted gray line); the CmR cells are not able to invade if their initial density lies below the threshold (shown by the dashed blue trajectory). However, after successful invasion (solid blue trajectory), the CmR cells can still be pushed below their critical density by competition with the CmS strain, triggering the collapse of both populations. (b), Dynamics of intracellular Cm concentrations and resource. Parameters are: r = 20.0, η = 0.85, kz = 4.0, c = 1.0, p = 50.0, hY = 0.25, kY = 2.5 and d = 30.0. (TIF) [file pbio.2000631.s005.tif]

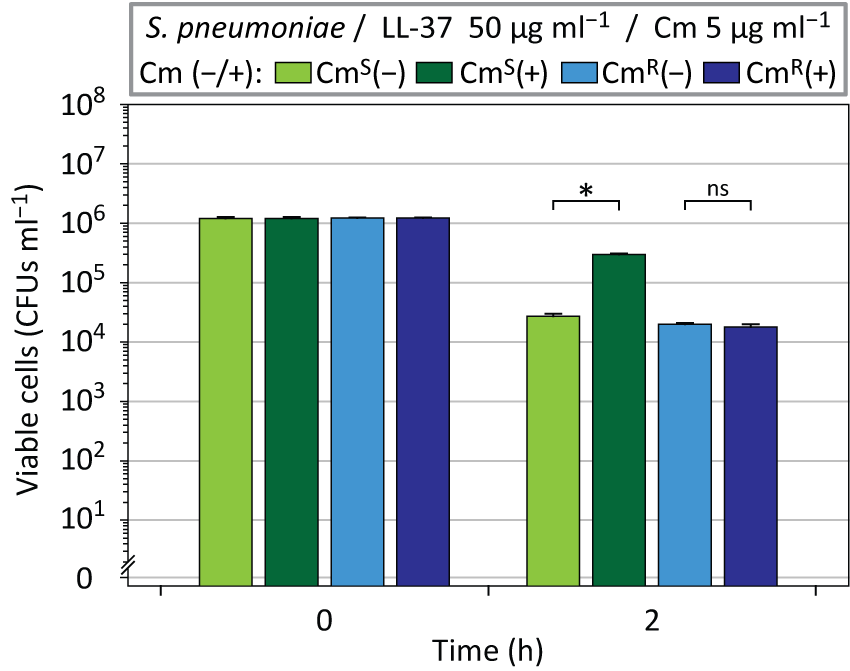

Supplement: S6 Fig — Killing of Cm-susceptible S. neumoniae D-PEP2K1 (CmS) and Cm-resistant D-PEP1C3 (CmR) by the human antimicrobial peptide LL-37 at a concentration of 50 μg ml−1, in absence (−) or presence (+) of 5 μg ml−1 chloramphenicol (Cm); average and s.e.m. of duplicates are shown. *P< 0.05; two-tailed t-test (see S1 Data). (TIF) [file pbio.2000631.s006.tif]
