# Supplementary material for: Collective Resistance in Microbial Communities by Intracellular Antibiotic Deactivation
Source: PLoS Biol. 2016 Dec 27;14(12):e2000631. doi: 10.1371/journal.pbio.2000631 (PMC5189934; doi:10.1371/journal.pbio.2000631)
Supplement: S1 Text — (PDF) [file pbio.2000631.s007.pdf]

S1 Text. Derivation of the mathematical model and model analysis.

S1.1. Model specification. We model a co-culture of genetically resistant (CAT-expressing; $\mathrm{Cm^R}$) and genetically susceptible ($\mathrm{Cm^S}$) bacterial cells, growing in the presence of chloramphenicol (Cm) in a chemostat environment. The two strains, which are assumed to be identical except for aspects directly associated with CAT expression, compete for a common limiting resource. The concentration of the resource, Z, changes through time according to the differential equation:

$$\frac{dZ}{dt} = \frac{Q}{V_{\mathrm{tot}}}(Z_0 - Z) - \frac{N_{\mathrm{s}}}{V_{\mathrm{tot}}}\mathrm{C}(Z, Y_{\mathrm{s}}) - \frac{N_{\mathrm{r}}}{V_{\mathrm{tot}}}\mathrm{C}(Z, Y_{\mathrm{r}}), \quad [1]$$

where Q is the flow rate of the chemostat, V_{tot} is its total volume, Z_0 is the concentration of the resource in the inflow medium, N_{s} and N_{r} denote the number of susceptible and resistant cells in the chemostat, and C is the per-capita resource-consumption rate, which is a function of the resource concentration and the intracellular concentrations of Cm in susceptible and resistant cells, Y_{s} and Y_{r}, respectively (see equation [4] below). The antibiotic also occurs in the medium, where its concentration is given by Y_{m}.

The following equations describe the change of the number of antibiotic molecules within cells and in the medium, due to inflow and outflow of the chemostat (of medium and cells), passive diffusion of Cm between medium and cell compartments, and degradation of the antibiotic in $\mathrm{Cm^R}$ cells

$$\begin{aligned}
\frac{d(V_{\mathrm{m}}Y_{\mathrm{m}})}{dt} &= Q\left(Y_0 - \frac{V_{\mathrm{m}}}{V_{\mathrm{tot}}}Y_{\mathrm{m}}\right) - PN_{\mathrm{s}}(Y_{\mathrm{m}} - Y_{\mathrm{s}}) - PN_{\mathrm{r}}(Y_{\mathrm{m}} - Y_{\mathrm{r}}),\\
\frac{d(vN_{\mathrm{s}}Y_{\mathrm{s}})}{dt} &= PN_{\mathrm{s}}(Y_{\mathrm{m}} - Y_{\mathrm{s}}) - \frac{Q}{V_{\mathrm{tot}}}vN_{\mathrm{s}}Y_{\mathrm{s}},\\
\frac{d(vN_{\mathrm{r}}Y_{\mathrm{r}})}{dt} &= PN_{\mathrm{r}}(Y_{\mathrm{m}} - Y_{\mathrm{r}}) - N_{\mathrm{r}}\mathrm{D}(Y_{\mathrm{r}}) - \frac{Q}{V_{\mathrm{tot}}}vN_{\mathrm{r}}Y_{\mathrm{r}}.
\end{aligned} \quad [2]$$

Here, v is the volume of a single cell, $V_{\mathrm{m}} = V_{\mathrm{tot}} - v\,(N_{\mathrm{s}} + N_{\mathrm{r}})$ is the volume of the medium, Y_0 is the concentration of Cm in the inflow medium, P measures the permeability of the cell membrane for Cm, and the function D quantifies the per-capita degradation rate of the antibiotic. We assume that the degradation of Cm by CAT follows Michaelis-Menten kinetics, i.e., $\mathrm{D}(Y) = d_{\max}\,Y/(K_{\mathrm{Y}} + Y)$, where $d_{\max}$ is the maximum degradation rate and K_{Y} is the half-saturation constant of CAT for Cm.

The growth of the two bacterial populations is assumed to be proportional to their respective rates of resource consumption, but we consider that CAT expression may have a negative effect on the growth rate of $\mathrm{Cm^R}$ cells. This potential cost is incorporated by allowing for different growth rate conversion factors g_{s} and g_{r} of the $\mathrm{Cm^S}$ and $\mathrm{Cm^R}$ cells. The population growth equations are thus given by

$$\begin{aligned}
\frac{dN_{\mathrm{s}}}{dt} &= g_{\mathrm{s}}\mathrm{C}(Z, Y_{\mathrm{s}})N_{\mathrm{s}} - \frac{Q}{V_{\mathrm{tot}}}N_{\mathrm{s}},\\
\frac{dN_{\mathrm{r}}}{dt} &= g_{\mathrm{r}}\mathrm{C}(Z, Y_{\mathrm{r}})N_{\mathrm{r}} - \frac{Q}{V_{\mathrm{tot}}}N_{\mathrm{r}}.
\end{aligned} \quad [3]$$

For simplicity, cell death is assumed to occur at a negligible rate, even in the presence of Cm, so cells are lost only through outflow from the chemostat. (This assumption can be relaxed, if necessary, by incorporating a positive rate of autolysis. The general analysis below, however,

shows that this has no qualitative effect on the conclusions, as long as the net growth rate decreases monotonically with the intracellular concentration of antibiotic).

The bacteriostatic effect of the antibiotic is modeled as a non-competitive inhibition of the resource consumption rate, C(*Z, Y*), which also depends on the resource concentration according to Michaelis-Menten kinetics. To be exact,

$$\mathrm{C}(Z,Y) = c_{\max}\frac{Z}{K_{\mathrm{Z}}+Z}\frac{H_{\mathrm{Y}}}{H_{\mathrm{Y}}+Y} \qquad [4]$$

where $c_{\max}$ is the maximum per-capita resource uptake rate in medium with a growth-saturating concentration of the resource and no antibiotic. All else being equal, cell growth is reduced by 50% relative to its value without antibiotic at Cm concentration $Y = H_{\mathrm{Y}}$. Similarly, half-saturated growth occurs at resource concentration $Z = K_{\mathrm{Z}}$.

S1.2. Rescaling. The model is next transformed into a dimensionless form, in order to reduce the number of free parameters. This rescaling step is accomplished by expressing the cell densities of Cm^{S} and Cm^{R} cells as volume fractions ($x_{\mathrm{s}} = N_{\mathrm{s}}\, v \,/\, V_{\mathrm{tot}}$ and $x_{\mathrm{r}} = N_{\mathrm{r}}\, v \,/\, V_{\mathrm{tot}}$), measuring time relative to the chemostat dilution time ($\tau = t\, Q \,/\, V_{\mathrm{tot}}$) and scaling the resource and Cm concentrations relative to their respective concentrations in the inflow medium ($z_{\mathrm{r}} = Z_{\mathrm{r}} / Z_0$, $y_{\mathrm{m}} = Y_{\mathrm{m}} / Y_0$, $y_{\mathrm{s}} = Y_{\mathrm{s}} / Y_0$, $y_{\mathrm{r}} = Y_{\mathrm{r}} / Y_0$). The resulting equations for the growth of the two bacterial populations are given by

$$\begin{aligned} \frac{dx_{\mathrm{s}}}{d\tau} &= x_{\mathrm{s}}\rho(z,y_{\mathrm{s}}) - x_{\mathrm{s}}, \\ \frac{dx_{\mathrm{r}}}{d\tau} &= \eta x_{\mathrm{r}}\rho(z,y_{\mathrm{r}}) - x_{\mathrm{r}}. \end{aligned} \qquad [5]$$

where

$$\rho(z,y) = r\frac{z}{k_{\mathrm{z}}+z}\frac{h_{\mathrm{y}}}{h_{\mathrm{y}}+y} \qquad [6]$$

is the scaled growth rate function, $r = g_{\mathrm{s}}\, c_{\max}\, V_{\mathrm{tot}} \,/\, Q$ is the (scaled) maximum growth rate of the Cm^{S} cells, and $k_{\mathrm{z}} = K_{\mathrm{Z}} / Z_0$ and $h_{\mathrm{y}} = H_{\mathrm{y}} / Y_0$, respectively, are the (scaled) half-saturation and inhibitory constants of the growth function. The parameter $\eta = g_{\mathrm{r}} / g_{\mathrm{s}}$ quantifies how efficiently the Cm^{R} strain grows relative to the susceptible one, which is a measure for the fitness cost of CAT expression.

For the resource concentration, we obtain

$$\frac{dz}{d\tau} = (1-z) - cx_{\mathrm{s}}\rho(z,y_{\mathrm{s}}) - cx_{\mathrm{r}}\rho(z,y_{\mathrm{r}})\,. \qquad [7]$$

Here, $c = 1 / (g_{\mathrm{s}}\, v\, Z_0)$ reflects the amount of resource needed to grow a volume unit of cells.

For the equations describing the Cm concentrations, we worked out the products in the derivatives on the left-hand side of equations [2]. The resulting equations,

$$\begin{aligned} \frac{dy_{\mathrm{m}}}{d\tau} &= \left(\frac{1}{1-x_{\mathrm{s}}-x_{\mathrm{r}}} - y_{\mathrm{m}}\right) - p\frac{x_{\mathrm{s}}(y_{\mathrm{m}}-y_{\mathrm{s}})+x_{\mathrm{r}}(y_{\mathrm{m}}-y_{\mathrm{r}})}{1-x_{\mathrm{s}}-x_{\mathrm{r}}} - y_{\mathrm{m}}\frac{d\ln(1-x_{\mathrm{s}}-x_{\mathrm{r}})}{d\tau}, \\ \frac{dy_{\mathrm{s}}}{d\tau} &= p(y_{\mathrm{m}}-y_{\mathrm{s}}) - y_{\mathrm{s}}\rho(z,y_{\mathrm{s}}), \\ \frac{dy_{\mathrm{r}}}{d\tau} &= p(y_{\mathrm{m}}-y_{\mathrm{r}}) - \delta(y_{\mathrm{r}}) - y_{\mathrm{r}}\eta\rho(z,y_{\mathrm{r}}), \end{aligned} \qquad [8]$$

have an additional term on the right-hand side that captures the effect of changes in medium volume and dilution of intracellular Cm due to cell growth. The rate of exchange between the compartments depends on the relative permeability $p = P V_{tot} / (v Q)$ of the cells to Cm. Finally, $\delta(y) = d y / (k_y + y)$, the rate of degradation of Cm by CAT, is characterized by two dimensionless parameters, a maximum rate $d = d_{max} V_{tot} / (v Y_0 Q)$, and a half-saturation constant $k_y = K_Y / Y_0$.

S1.3. Qualitative model analysis. In order to characterize the conditions that allow for stable coexistence between the two strains (e.g., Fig 4), we next perform a qualitative equilibrium stability analysis of the model (equations [5] – [8]). Accordingly, we suppose that the Cm^S and Cm^R cells attain equilibrium densities of x_s^* and x_r^*, respectively, and then ask under what conditions this equilibrium exists and is stable.

At equilibrium, the two net population growth rates must be zero, so that we obtain a first equilibrium condition from equation [5]:

$$\rho(z^*, y_s^*) = \eta\rho(z^*, y_r^*) = 1. \quad [9]$$

This condition states that the loss of cells from the chemostat must be balanced by cell growth for both cell types. Here (and throughout), equilibrium values of variables are marked with an asterisk. Based on equality [9], the equilibrium conditions for the intracellular concentrations of Cm (equations [8]; $dy_s/d\tau = 0$ and $dy_r/d\tau = 0$) lead to the obvious result that

$$y_r^* = \frac{p y_m^* - \delta(y_r^*)}{p+1} < \frac{p y_m^*}{p+1} = y_s^* \quad \text{for any } y_r^* > 0, \quad [10]$$

i.e., the intracellular concentration of Cm in Cm^R cells is lower than in Cm^S cells, given that $\delta(y_r^*) > 0$ for any $y_r^* > 0$.

Given that Cm inhibits cell growth ($\rho(z, y)$ is a monotonically decreasing function of y, condition [10] implies that $\rho(z^*, y_s^*) < \rho(z^*, y_r^*)$. Hence, condition [9] can only be satisfied if

$$\eta = \frac{\rho(z^*, y_s^*)}{\rho(z^*, y_r^*)} < 1. \quad [11]$$

From this general result, we conclude that coexistence between Cm^S and Cm^R strains can be achieved only when CAT expression is costly.

In order to assess the stability of the coexistence equilibrium, we must take into consideration the ecological feedback between the bacterial populations and their environment, specifically, the concentration of Cm in the medium. If this environmental feedback is weak, y_s^* and y_r^* will vary only little with the relative frequencies of the two strains, implying that condition [9] can be satisfied for only a limited range of values for η. Conversely, robust coexistence relies on a strong environmental feedback, in which the Cm^R cells must exert a high level of control over the extracellular concentration of chloramphenicol. This requires that the strain must be able to reach a high population density. In a well-mixed chemostat model, coexistence is therefore observed only when the cell volume fractions reach high values (>20%); however, in spatially structured environments, cells can have a large influence on their local environment even if the overall population size is small.

S1.4. Numerical bifurcation analysis. While a strong environmental feedback is necessary for robust coexistence, such coexistence must also be dynamically stable. To evaluate the stability of coexistence between Cm^S and Cm^R strains, we performed extensive numerical simulations across a range of parameter conditions and classified the dynamics of the model based on the number and the stability of the equilibria that were observed in these simulations (S4 Fig). One general observation across the whole set of simulations is that if interior equilibria ($x_s^* > 0$ and $x_r^* > 0$) are present, always at least one of them is stable. In particular, there was no parameter condition for which we found mutual exclusion, i.e., an unstable interior equilibrium from which small departures lead to a population dominated by either one or the other cell type depending on the initial abundances.

These results are explained by the fact that the marginal impact of Cm^R cells on the chemostat environment diminishes as the Cm^R strain becomes more abundant. Figure 4b shows clearly why this is the case: the invasion of Cm^R cells causes a reduction in the intra- (and extra-) cellular Cm concentrations, strongly decreasing the level of antibiotic stress for both types of cells. At lower stress levels, the relative advantage of Cm degradation is lower as well. As the Cm^R population continues to grow, the relative growth rate bonus of Cm^R cells may eventually become so low that it is exactly balanced by the cost of CAT expression. When that happens, stable coexistence between Cm^R and Cm^S strains can be maintained dynamically, satisfying equilibrium condition [9].

The frequency-dependent effect of Cm^R cells on the environment also explains why we observe bistability in a large region of the parameter space (areas between dashed and solid blue lines in S4 Fig). In this area, a population of Cm^R cells can maintain itself at high density, but cannot invade an empty chemostat inoculated with a few individuals. Here, a large population of Cm^R cells is necessary to reduce the Cm concentration below the critical concentration that still permits persistent growth in the chemostat.

Given that the Cm^S strain is a superior competitor, it is possible for that strain to push the Cm^R cells below their critical density. When this happens, and the Cm^S strain cannot survive on its own, we observe competition-induced extinction (S5 Fig). In this regime, a population of Cm^R cells is first invaded by the susceptible strain and eventually outcompeted. The remaining Cm^S cells, however, cannot survive on their own, as the concentration of antibiotic is no longer kept low by the resistant cells. This counter-intuitive process is an example of the 'resident-strikes-back' phenomenon (Mylius & Diekmann, 2001), which has received previous attention in the theoretical literature.

S1 Text Reference:

Mylius SD, Diekmann O. The resident strikes back: invader-induced switching of resident attractor. J. Theor. Biol. 211: 297-311 (2001).
